# The Use of Compost and Arbuscular Mycorrhizal Fungi and Their Combination to Improve Tomato Tolerance to Salt Stress

**DOI:** 10.3390/plants13162225

**Published:** 2024-08-11

**Authors:** Fadoua Mekkaoui, Mohamed Ait-El-Mokhtar, Nada Zaari Jabri, Ilham Amghar, Soukaina Essadssi, Abdelaziz Hmyene

**Affiliations:** Laboratory of Biochemistry, Environment, and Agri-Food URAC 36, Department of Biology, Faculty of Science and Techniques—Mohammedia, Hassan II University, Mohammedia 28806, Morocco; fadouamekkaoui1@gmail.com (F.M.); nadazaarijabri@gmail.com (N.Z.J.); amghar.ilham@gmail.com (I.A.); soukainaessadssi@gmail.com (S.E.); hmyeneaziz2002@yahoo.fr (A.H.)

**Keywords:** antioxidant activity, arbuscular mycorrhizal fungi, biostimulants, compost, oxidative stress, plant tolerance, salinity, tomato

## Abstract

Salinity poses a significant challenge to tomato plant development and metabolism. This study explores the use of biostimulants as eco-friendly strategies to enhance tomato plant tolerance to salinity. Conducted in a greenhouse, the research focuses on the *Solanum lycopersicum* L. behavior under saline conditions. Tomato seeds were treated with arbuscular mycorrhizal fungi (AMF), compost, and their combination under both non-saline and saline conditions (0 and 150 mM NaCl). Plant height, number of flowers and fruits, shoot fresh weight, and root dry weight were negatively impacted by salt stress. The supplementation with compost affected the colonization of AMF, but the application of stress had no effect on this trait. However, the use of compost and AMF separately or in combination showed positive effects on the measured parameters. At the physiological level, compost played a beneficial role in increasing photosynthetic efficiency, whether or not plants were subjected to salinity. In addition, the application of these biostimulants led to an increase in nitrogen content in the plants, irrespective of the stress conditions. AMF and compost, applied alone or in combination, showed positive effects on photosynthetic pigment concentrations and protein content. Under salt stress, characterized by an increase in lipid peroxidation and H_2_O_2_ content, the application of these biostimulants succeeded in reducing both these parameters in affected plants through exhibiting an increase in antioxidant enzyme activity. In conclusion, incorporating compost, AMF, or their combined application emerges as a promising approach to alleviate the detrimental impacts of salt stress on both plant performances. These findings indicate optimistic possibilities for advancing sustainable and resilient agricultural practices.

## 1. Introduction

Climate change is among the main encounters facing the world today. Agriculture, being a crucial sector, is likely to be strongly impacted by this phenomenon. This situation has significant social and economic implications for human well-being. By 2050, global food needs are expected to expand from 35 to 56% due to projected population growth, exceeding 9 billion people [1].

Soil salinization is a closely related phenomenon to climate change and a major problem that affects and decreases global food production, jeopardizing land productivity. It is estimated that by 2050, 50% of all arable land will be impacted by salinity [2]. This is the result of the accumulation of water-soluble salts in the soil, including sulphate, chloride, carbonate, bicarbonate, potassium, sodium, and magnesium [3,4]. Soil salinity is affected by multiple factors, including drought, water shortages, and the use of poor-quality water for irrigation [5]. This salinity leads to physiological abnormalities that negatively affect plant development and growth. Consequences include a decreased leaf development rate, decreased cell elongation and division, and lower intensity of photosynthesis [6]. In addition, soil salinity limits seed germination and stomata opening, reducing transpiration and CO_2_ uptake [7,8]. Several researchers have pointed out that soil salt affects crop growth and productivity, including vegetables, which are known for their sensitivity to salinity [9]

Soil salinity can induce three consecutive stresses inside the plant: osmotic stress, ionic stress, and oxidative stress [10,11]. Among the water-soluble salts responsible for salinity, sodium (Na) and chlorine (Cl) ions are considered the main contributors, where the excess of Na^+^ contributes to sodicity among the exchangeable cations [4]. Salinity promptly induces reactive oxygen species (ROS) and oxidative damage. Despite their harmfulness at high concentrations for plants, ROS act as signaling molecules at low concentrations. Plant cells perceive these high levels of ROS and quickly trigger their elimination by trapping them, thus inducing an adaptive response [12]. To prevent ROS-related damage in salt-affected plants, various enzymatic and non-enzymatic antioxidant eliminators are used. Enzymatic eliminators include superoxide dismutase (SOD), catalase (CAT), ascorbate peroxidase (APX), polyphenol oxidase (PPO), peroxidase (POX), dehydroascorbate reductase (DHAR), peroxiredoxins (PRXs), glutathione peroxidase (GPX), glutathione reductase (GR), thioredoxin (TRX), glutathione S-transferase (GST), and monodehydrohascorbate reductase (MDHAR), while non-aenzymatic eliminators include glutathione (GSH), ascorbate (AsA), alkaloids, carotenoids, phenols, flavonoids, non-protein amino acids, and tocopherol [13,14,15,16].

Innovative biological advances provide practical and necessary ways to improve plant tolerance to salinity, complementing adaptive mechanisms developed by plants. Recent research strategies promoting the boosting effects of various organic fertilizers on plant performances in salt-affected soils have reported a lessening of osmotic and oxidative stress, improved stomatal conductance and density, as well as an improvement in seed germination rate and the promotion of increased microbial activities [17,18]. The use of organic materials such as compost has shown significant benefits in improving the microbiome of saline soil. In addition, compost supplementation may alleviate the adverse impact of salinity in plants by promoting soil quality; enhancing nutrient availability for plants; and boosting various metabolic processes, such as photosynthesis and respiration and the antioxidant system [19]. Numerous studies have examined the application of compost in the restoration of salt-affected soils, confirming its effectiveness [20,21]. 

There is growing scientific awareness of the role of mycorrhizae in plant responses to environmental stresses. Mycorrhiza is a type of mutualist relationship established between a fungus and more than 80% of plants on earth, and it was first described by Frank in 1885 [22]. In this association, the plant provides arbuscular mycorrhizal fungi (AMF) with a source of carbon and a place to live, and in exchange, AMF provides water and essential mineral elements. AMF can maintain approximately 90% of the plant’s phosphorus (P) and 60% of its nitrogen (N) [23]. After the establishment of symbiosis, mycorrhizal plants utilize two pathways for nutrient uptake. They can directly absorb nutrients from the soil through root hairs and the root epidermis or indirectly acquire nutrients through the AMF hyphae at the interface between the plant and the fungus. According to Dai et al. [24], *Glomus* species increased P and N uptake in organic field wheat by nearly 2.3 times compared to the typical method. The nature of AMF may be largely altered by soil, plant, and fungal conditions [25]. The establishment of the association between AMF and plants is very complex and requires communication via various chemical molecules that lead to common recognition and the establishment of symbiotic-related structures. Myc factor, derived from the fungus, and strigolactones, produced by plant roots, are the two major components orchestrating the implementation of the symbiosis. Once the plant emits the chemical signals, AMF identify it, and the expansion of hyphae towards plant roots is triggered to form an appressorium at the root surface [22].

Tomatoes (*Solanum lycopersicum* L.) are commonly recognized as an essential vegetable. They are a common ingredient in many dishes around the world [26]. Tomatoes are rich in nutrients and bioactive substances that are essential for human health, weight loss, and healthy skin, and can improve or avoid multiple long-lasting degenerative diseases [27,28]. They contain carotenoids, including lycopene and beta-carotenoids; tocopherol (vitamin E); ascorbic acid (vitamin C); and bioactive phenolic molecules such as kaempferol, quercetin, lutein, naringenin, and ferulic, chlorogenic, and caffeic acids [28,29,30]. These compounds are known to have anticancer and antioxidant properties [29,31]. 

Tomato cultivation is moderately salt-sensitive and needs regular and constant watering as well as suitable nutrients for optimal development and yield. Soil and groundwater salinization is among the main issues hampering the production of tomato in many countries around the world, particularly in the Mediterranean region [32]. Furthermore, it is important to note that sensitivity to salinity can vary significantly between different tomato varieties [33]. To date, several studies have been carried out to better understand the effect of saline stress on the response of tomatoes to this stress as well as the application of compost [34,35] and AMF [36,37]; however, no study has looked at the effect of the combination of compost and AMF on tomato tolerance to salt stress. The present research was performed to study the role of indigenous AMF and compost, applied separately or in combination, on tomato’s morphological, physiological, and biochemical traits. This study hypothesized that the application of biological stimulants, specifically AMF and compost, could enhance tomato plants’ resilience to salt stress by boosting their defense mechanisms. In addition, the goal of this study was to investigate the individual and synergistic effects of AMF and compost on the salt stress tolerance of tomato plants.

## 2. Results

### 2.1. Growth, Yield, and Mycorrhizal Colonization Traits

Salt stress had a significant negative impact on several aspects of plant growth (Figure S1), yield, and physiology (Table 1), including plant height (PH), number of leaves (NL), number of fruits (NFr), shoot fresh weight (SFW), and root dry weight (RDW), as well as photosynthetic efficiency (Fv/Fm), with decreases of 17% for PH, 30% for NL, 58% for NFr, 32% for SFW, 65% for RDW, and 10% for Fv/Fm compared to the non-stressed control. Salinity stress did not have any significant effect on root elongation, leaf number, flower number, shoot dry matter, or stomatal conductance. The application of AMF had a positive effect on the number of leaves and flowers, with increases of 50 and 429%, respectively, while the application of AMF+compost influenced the number of fruits, with an increase of 125% compared to the stressed control. The applied biostimulants had a positive effect on Fv/Fm (12% increase for C, 7% increase for M, and 10% increase for CM) compared to the control under 150 mM NaCl. The control plants showed no root colonization by AMF. Furthermore, the addition of compost resulted in a reduction in mycorrhizal parameters compared to unamended AMF treatments, especially under saline stress (51 and 92% decreases for Fa and Ma, respectively). In addition, it should be noted that saline stress resulted in a significant increase in colonization intensity in plants inoculated with AMF compared to those not exposed to stress, with an increase of 38%.

### 2.2. Photosynthetic Pigments Content

**Figure 1:**
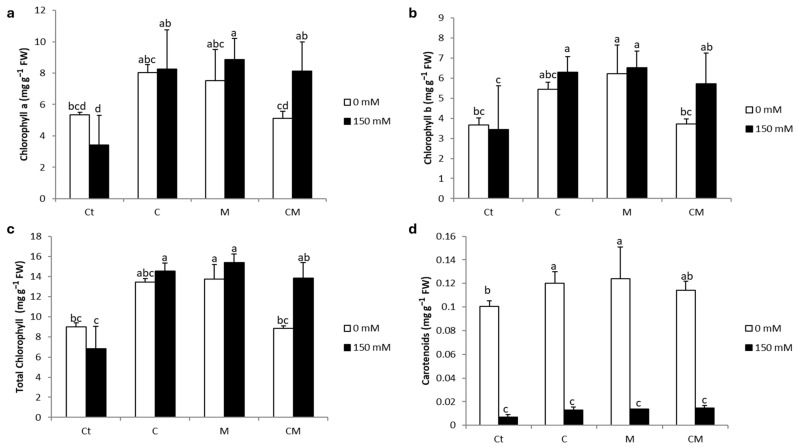
Photosynthetic pigment ((**a**) chlorophyll *a*, (**b**) chlorophyll *b*, (**c**) total chlorophyll, (**d**) carotenoids) content of tomato plants under saline and non-saline conditions after the application of compost and AMF alone or in combination. Ct: control; C: compost; M: arbuscular mycorrhizal fungi; CM: combination of compost and arbuscular mycorrhizal fungi. Data are means of three replicates (n = 3) ± standard error (SE). The bars of each parameter labeled by different letters indicate significant differences assessed by Duncan’s test after performing a three-way ANOVA (*p* < 0.05).

The results shown in Figure 1 indicate that the application of salt stress resulted in a decrease in chlorophyll concentration, where chlorophyll *a* decreased by 36%, chlorophyll *b* by 6%, and carotenoids suffered a whopping decrease of 93%. However, the application of biostimulants positively counteracted the negative effect of salt stress and stimulated the concentration of total chlorophyll and chlorophyll *a* and *b*. The highest values of improvement were recorded for the M treatment, with 125, 160, and 90%, respectively. 

### 2.3. Nitrogen Content

**Figure 2:**
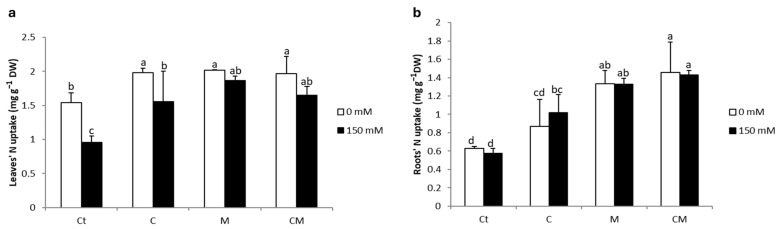
Nitrogen content of tomato leaves (**a**) and roots (**b**) under saline and non-saline conditions after the application of compost and AMF alone or in combination. Ct: control; C: compost; M: arbuscular mycorrhizal fungi; CM: combination of compost and arbuscular mycorrhizal fungi. Data are means of three replicates (n = 3) ± standard error (SE). The bars of each parameter labeled by different letters indicate significant differences assessed by Duncan’s test after performing a three-way ANOVA (*p* < 0.05).

Salt stress had a significant effect on the nitrogen content in leaves and roots, resulting in decreases of 38 and 8% respectively (Figure 2). However, stressed plants treated with C, M, and CM showed significant increases in nitrogen content in the leaves and roots compared to the stressed control. The highest value for leaves was recorded for the M treatment, with 95%, and CM for the roots, with 147%.

### 2.4. Hydrogen Peroxide and Malondialdehyde Content

**Figure 3:**
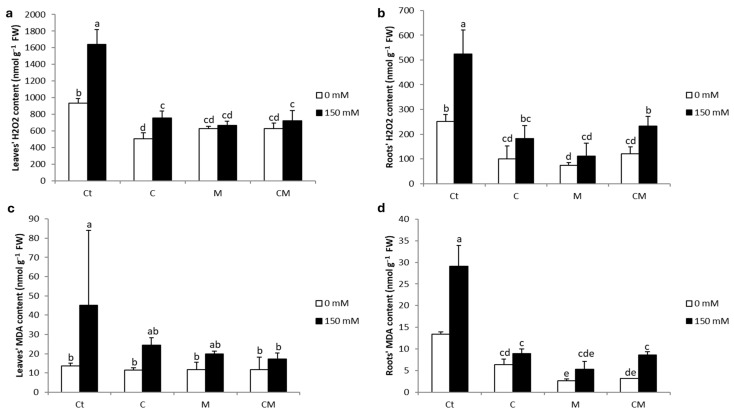
Hydrogen peroxide (**a**,**b**) and malondialdehyde (**c**,**d**) contents in the shoot and the root of tomato plants under saline and non-saline conditions after the application of compost and AMF alone or in combination. Ct: control; C: compost; M: arbuscular mycorrhizal fungi; CM: combination of compost and arbuscular mycorrhizal fungi. Data are means of three replicates (n = 3) ± standard error (SE). The bars of each parameter labeled by different letters indicate significant differences assessed by Duncan’s test after performing three-way ANOVA (*p* < 0.05).

The significant increase in H_2_O_2_ and MDA levels in saline-stressed plant tissues compared to the control (Figure 3) highlights their role as indicators of oxidative stress. H_2_O_2_ levels significantly rose by 76 and 108% in leaves and roots, respectively, while MDA levels increased by 228 and 117% in the same parts of the plant, respectively. The application of the biostimulants significantly reduced both parameters under salt stress. In leaves, the observed reductions were 54, 60, and 56% for H_2_O_2_ concentration and 46, 56, and 62% for MDA concentration under C, M, and CM treatments, respectively. In roots, the reductions were even more pronounced, with decreases of 56, 79, and 51% for H_2_O_2_ content and 69, 82, and 71% for MDA content for C, M, and CM treatments, respectively. These results demonstrate the effectiveness of biostimulants in reducing oxidative stress in plant tissues under salt stress.

### 2.5. Soluble Sugar and Protein Content

**Figure 4:**
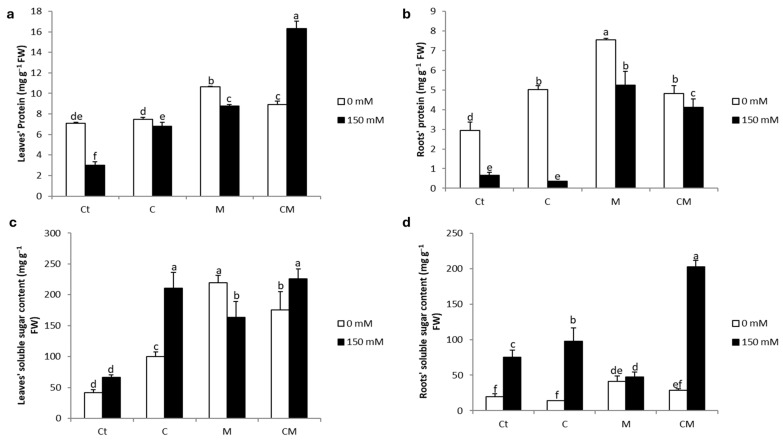
Protein (**a**,**b**) and soluble sugar (**c**,**d**) contents in the shoots and the roots of tomato plants under saline and non-saline conditions after the application of compost and AMF alone or in combination. Ct: control; C: compost; M: arbuscular mycorrhizal fungi; CM: combination of compost and arbuscular mycorrhizal fungi. Data are means of three replicates (n = 3) ± standard error (SE). The bars of each parameter labeled by different letters indicate significant differences assessed by Duncan’s test after performing three-way ANOVA (*p* < 0.05).

The data from Figure 4 demonstrates the impact of salt stress and different treatments on the soluble sugar concentration. The application of stress resulted in a significant increase in the concentration of soluble sugar at the root level, with an increase of 278%. On the other hand, no significant increase was observed at the leaf level. Under stress conditions, plants treated with the different treatments (C, M, and CM) showed significantly higher soluble sugar concentrations in the leaves compared to the non-stressed control plants. The improvements were 217, 146, and 240%, respectively, for the C, M, and CM treatments. At the level of roots under stress, a significant increase was recorded in plants treated with C and CM treatments, with increases of 29 and 168%, respectively.

Plants subjected to salinity showed a significant decrease in protein concentration compared to non-stressed control plants, both at the leaf and root levels, with 58 and 77%, respectively (Figure 4). However, the application of the biostimulants under stress resulted in a significant increase in protein content in the leaves compared to the stressed control. On the other hand, at the root level, this increase was only observed in the M and CM treatments in the presence of stress, with 687 and 517% increases, respectively, compared to the stressed control.

### 2.6. Antioxidant Enzyme Activity

Stress significantly increased CAT activity content in the stressed controls compared to the non-stressed controls in both the leaves and roots, with 143 and 92%, respectively (Table 2 and Table 3). In addition, the application of biostimulants further significantly increased CAT activity under stress compared to the stressed controls, specifically in the leaves with 53%. For PPO and POX activity, stress did not have a significant effect on the controls compared to the unstressed ones, either in the leaves or roots. However, the application of C, M, and CM treatments under stress significantly increased PPO and POX activity in the leaves compared to the controls. On the other hand, at the root level, only C treatment showed a significant effect on the activity of PPO and POX, unlike the other treatments with 114 and 17%, respectively. The exposure to stress and the application of biostimulants did not have a significant impact on the activity of APX in the leaves. However, the application of compost under stress conditions resulted in a noticeable increase in roots’ APX activity, with 132%.

### 2.7. Principal Component Analysis

**Figure 5:**
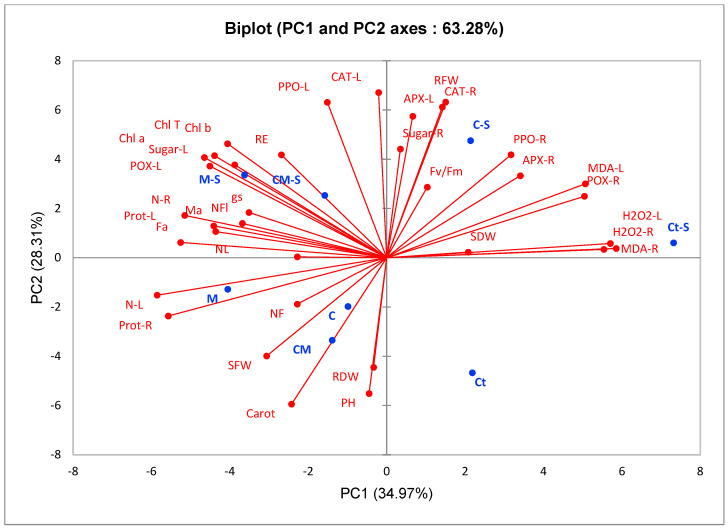
Principal component analysis of the studied traits of different biostimulant treatments in tomato plants growing under saline and non-saline conditions. S: salinity; Ct: control; C: compost; M: arbuscular mycorrhizal fungi; CM: combination of compost and arbuscular mycorrhizal fungi; Ct-S: control+stress conditions; C-S: compost+stress conditions; M-S: arbuscular mycorrhizal fungi+stress conditions, CM-S: combination of compost and arbuscular mycorrhizal fungi+stress conditions; PH: plant height; NL: number of leaves; SDW: shoot dry weight; RDW: root dry weight; Fa: AMF infection frequency; Ma: AMF infection intensity; gs: stomatal conductance; Fv/Fm: photosynthetic efficiency; Chl *a*: chlorophyll *a*; Chl *b*: chlorophyll *b*; Chl T: total chlorophyll; Carot: carotenoids; H_2_O_2_-L: leaves H_2_O_2_ content; H_2_O_2_-R: root H_2_O_2_ content; MDA-L: leaves MDA content; MDA-R: root MDA content; Sugar-L: leaf sugar content; Sugar-R: root sugar content; APX-L: leaf ascorbate peroxidase activity; APX-R: root ascorbate peroxidase activity; CAT-L: leaf catalase activity; CAT-R: root catalase activity; POX-L: leaf peroxidase activity; POX-R: root peroxidase activity; PPO-L: leaf polyphenol oxidase activity; PPO-R: root polyphenol oxidase activity; Prot-L: leaf protein content; Prot-R: root protein content.

A principal component analysis (PCA) was performed to determine the interactions between the measured parameters and the applied treatments. The data showed two main components: PC1 accounted for 34.97% and PC2 accounted for 28.31% (Figure 5). The PCA analysis revealed a positive correlation between compost and/or AMF biostimulants under non-saline conditions for NF, PH, SFW, RDW, and leaf nitrogen (N-L) content, as well as carotenoid (carot) and protein content at the root level (Prot-R), which were strongly correlated, while PCA confirmed the negative effect of salt stress on these parameters. Additionally, the PCA showed a positive correlation between the application of stress and the H_2_O_2_ and MDA contents. When applying AMF alone or with compost under salt stress, the analysis showed a positive correlation between these two treatments for NL, NFl, RE, and gs, as well as total chlorophyll (Chl T), chlorophyll *a* (Chl *a*), chlorophyll *b* (Chl *b*) content, AMF infection, protein and soluble sugar content, and antioxidant activity at the leaf level (CAT-L, PPO-L, and POX-L). The biplot also revealed a positive correlation between compost application under salt stress, soluble sugar (Sugar-R) content, and antioxidant activity in the roots (CAT-R, PPO-R, POX-R and APX-R), FRW, and Fv/Fm.

## 3. Discussion

Recently, biostimulants have attracted increasing attention as an alternative to intensive agriculture and chemical fertilizers. Among these alternatives, AMF and compost stand out, since they offer promising potential to be integrated into sustainable agriculture, aiming to preserve and improve soil fertility while ensuring long-term agricultural production. The main aim of this study was to examine the impact and effectiveness of AMF, compost, and their combination on tomato growth, yield, and physiological and biochemical traits in a saline environment (150 mM NaCl). 

In this research, a clear decline in tomato plant growth indicators was observed under salinity conditions (Table 1). The assessment of plant salt tolerance, as mentioned by da Silva et al. [38], is often based on the biomass produced. Despite the general trend that the root parts of plants are less affected by excess salt than their aerial parts, as noted by Munns and Tester, a greater vulnerability of root dry weight to salinity compared to that of leaves was revealed in this study [39]. This peculiarity can be attributed to the predisposition of the roots to be the first to face the accumulation of salt in the soil solution [40]. A crucial aspect of root systems compared to aerial parts lies in their anatomy. In the roots, the tissues of the stele are surrounded by the cortex and the epidermis, while in the aerial parts, they are distributed through the parenchymal cells. There is evidence that the root cortex must primarily exclude Na^+^ from aerial parts, requiring energy expenditure (ATP) used by antiporters as part of tissue tolerance [41].

In addition, a negative influence of compost addition on mycorrhizal colonization was shown by the current study (Table 1), and this finding is consistent with the results of previous research [42,43,44]. This observation may be attributed to the presence of organic decomposition products in compost, which can contain substances that inhibit root colonization with AMF. These decomposition by-products may create an environment that is less conducive to AMF colonization, thereby decreasing their ability to colonize plant roots [45]. Contrary to what could be expected, the application of salt stress did not have a negative effect on mycorrhizal colonization of plants treated only with AMF. This could be explained by the fact that AMF were taken from areas of saline soil and are most effective when exposed to the presence of salts. 

It has been observed that salt stress induces a decrease in nitrogen content both in the leaves and roots (Figure 2), a phenomenon that can be explained by a reduction in the expression of key genes involved in ammonium (NH_4_^+^) assimilation as well as nitrate reductase (OsNR1). This decrease in gene expression hinders the ability of plants to convert nitrates (NO_3_^-^) to NH_4_^+^, thus reducing the supply of NH_4_^+^, which is essential for many metabolic processes [46]. The favorable impact of compost addition can be explained by its richness in nitrogen, an essential element of various enzymes involved in photosynthesis, including RuBisCO [47]. In addition, the application of compost can promote the accumulation of betaine glycine and proline in seedlings, compounds that stabilize enzymes involved in CO_2_ fixation, such as RuBisCO and carbon anhydrase [47]. This accumulation also helps protect the pigment–protein complexes related to PSII [47,48]. Similarly, increased mycorrhizal colonization under stress was associated with increased nitrogen fixation. These observations are in agreement with the results of previous research that has also highlighted the positive effects of AMF on plant growth, nitrogen balance index, and the expression of genes involved in salt stress [36]. 

Gas exchange characteristics are frequently used to assess the overall performance of plants. Salt stress results in damage to the thylakoid membrane, which hosts a diversity of photosynthetic pigments, causing an initial alteration of the photosynthetic machinery, followed by deterioration in plant growth and crop productivity [49]. This salinity can impact photosynthesis directly or indirectly by reducing the availability of CO_2_, notably by diffusion limitations. Under saline stress, photosynthesis may be hampered either by a decrease in stomatal conductance or by other non-stomatal factors such as a reduction in chlorophyll pigments required for light absorption [49]. In this study, the saline condition caused a decrease in F_v_/F_m_ and an increase in chlorophyll degradation (Table 1 and Figure 1). The decrease in pigment content observed in untreated stressed plants can be attributed not only to an increase in degradation, but also to an inhibition of chlorophyll synthesis induced by salinity. Similar observations have been reported in studies on alfalfa [50], carob [51], and date palm [42]. Plants treated with AMF and/or enriched with compost showed increased photosystem II efficiency, as well as a higher concentration of photosynthetic pigments compared to the control group; this indicates that the application of both biostimulants improved the physiological state of plants subjected to saline condition. These improvements can promote an increase in the rate of photosynthesis by facilitating a better assimilation of CO_2_. This phenomenon could explain the increase in the growth of tomato plants and the reduction in the inhibitory effect of salinity on physiological parameters. In a similar vein, Anli et al. [52] hypothesized that the combined treatment of organic amendment and AMF prevents abiotic stress from disrupting photosynthesis by improving the synthesis of chlorophyll pigments. The results of this study highlighted the synergistic effect of compost and AMF on the photosynthetic characteristics of tomatoes. The improvement of chlorophyll synthesis is closely related to the absorption, efficiency, and transport of mineral elements, which are essential for the formation of heme and the synthesis of chlorophyll, and act as co-factors in various critical cellular processes, including photosynthesis. Therefore, this combination could offer tomato plants a better absorption capacity of nutrients and phytohormones necessary to increase the efficiency of photosynthesis and promote plant growth under salt stress conditions [43].

The observations of the present study have shown that exposure to saline stress leads to an increase in lipid peroxidation and H_2_O_2_ concentration (Figure 3) in tomato plants, which is consistent with previous studies on tomatoes [53,54] and wheat [55,56], as well as on quinoa [21]. Following the neutralization of superoxide radicals, plants exposed to salt produce H_2_O_2_, a toxic compound that can cause damage to plants. High H_2_O_2_ concentrations result in lipid peroxidation and damage to cell membranes [53,57]. In this study, tomato plants grown under 150 mM NaCl in the presence of AMF, compost, and their combination showed lower concentrations of MDA and H_2_O_2_ compared to untreated stressed plants (Figure 3). Similar results have been obtained in previous studies conducted by Ait-El-Mokhtar et al. [42] as well as by Ben-Laouane et al. [58] on date palm and alfalfa. In addition, in this study, the application of biostimulants resulted in a reduced degree of oxidative damage, as evidenced by lower levels of H_2_O_2_ and MDA. This reduction could protect the photosynthetic machinery from oxidative damage induced by salt stress and improve the concentration of proteins and osmolytes [59]. This protection could be attributed to the improvement of the antioxidant enzymatic system, which eliminates ROS before they damage membrane lipids, thereby reducing lipid peroxidation [60]. This decrease in the concentration of these lesions results from increased activity of antioxidant enzymes and microorganism-induced trapping genes [61] and/or compost use [62]. Previous research has highlighted that cell membrane resistance is enhanced by proteins, which helps to reduce membrane unsaturation [63]. These observations were corroborated by the results obtained. In this study, the application of compost in association with the inoculation of AMF consortium, either in individual or combined treatment, has been shown to be very effective in increasing protein concentration in tomato plant leaves compared to the control plants not treated with compost or microorganisms. These findings are consistent with previous studies using AMF or plant-growth-promoting rhizobacteria (PGPR) [52].

In this study, a significant increase in the concentration of soluble sugars was observed in the leaves of stressed tomato plants, an increase that was further accentuated in the presence of AMF and/or compost (Figure 4). On the other hand, the separate application of AMF had no significant impact on this trait at the roots level. Soluble sugars, known as organic osmolytes, are widely used to assess the response to salt stress [59]. Several research studies have highlighted the positive role of carbohydrates in regulating the osmotic potential of cells, thus facilitating the absorption of water under adverse conditions [40,56,64]. This response could aim to maintain adequate levels of hydration and turgor, thus ensuring the proper functioning of physiological activities in stressful environments [64,65]. 

Antioxidant enzymes are critically important in the elimination of ROS and in limiting damage caused by oxidative stress [66]. Activation of ROS-trapping enzymes, such as POX and PPO, is the predominant mechanism of cell detoxification under stress [60,67]. Thus, the ability of plants to tolerate salinity is closely related to the induction of an antioxidant enzyme system and the limitation of oxidative damage [68], an activity that is reinforced in plants treated with AMF and/or compost. SOD is recognized as one of the first lines of enzyme defense, catalyzing the conversion of superoxide radicals to hydrogen peroxide, which is then neutralized into water [69]. This transformation of H_2_O_2_ to H_2_O can be promoted by the action of other enzymes such as CAT, POX, and PPO [69]. In this study, the increased activity of hydrogen peroxide-trapping enzymes (CAT, POX, and PPO) demonstrates the presence of SOD activity. Moreover, the increase in antioxidant enzyme activity in plants treated with AMF and compost compared to untreated plants (Table 2 and Table 3) was associated with a decrease in H_2_O_2_ content and lipid peroxidation, suggesting an effective system for neutralizing ROS in tomato plants, resulting in reduced oxidative stress and reduced membrane damage in treated plants [42]. 

**Figure 6:**
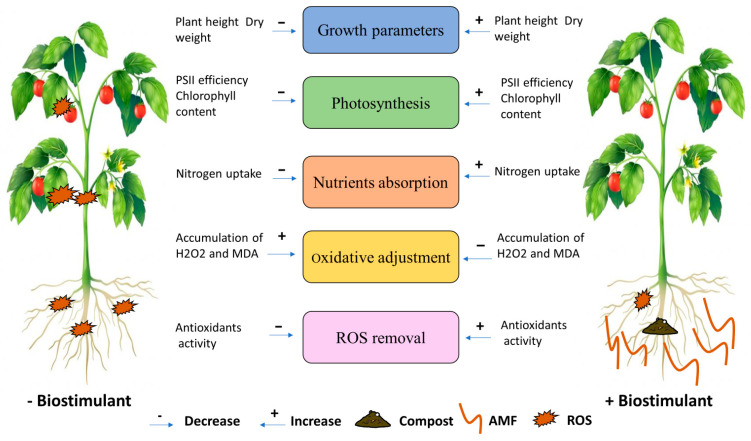
Schematic representation of various mechanisms induced by AMF and compost application in tomato plants under salt stress.

Overall, the results show that the application of compost and/or AMF synergistically reduced salt stress in tomato crops, acting through physiological and biochemical processes (Figure 6). Although organic amendment resulted in a decrease in root colonization by AMF, the combined effect of compost and AMF as well as AMF applied alone appear to be the most effective way to increase tomato seedlings’ tolerance to salinity. Indeed, the application of AMF and/or compost appears to confer several forms of protection to tomato plants, with these applications being positively correlated with growth parameters, photosynthetic plant characteristics, and nutrient homeostasis. AMF, alone or in combination with compost, were negatively associated with MDA and H_2_O_2_ content. Under stress, the application of AMF and/or compost was positively correlated with the activity of antioxidant enzymes (CAT, PPO, and POX) and the biosynthesis of proteins and soluble sugars. These correlations suggest that the application of microbial inoculum and compost coincided with an improvement in foliar gas exchange, the induction of the photosynthetic apparatus and an increased accumulation of pigments associated with light collection, and the mitigation of stress damage by regulating antioxidant enzymes under high salinity [42].

## 4. Materials and Methods

### 4.1. Plant and Biostimulant Materials and Experimental Design 

*Solanum lycopersicum* L. seeds, Cambell33 variety, were surface-sterilized with 10% bleach for 10 min and then rinsed four times with sterile distilled water. The seeds were germinated in Petri dishes containing filter paper moistened with 2 mL of distilled water and were incubated for seven days at a temperature of 25 °C. Then, the seeds were transplanted into alveoli containing peat for 21 days, then transferred to plastic seed trays containing 1.6 kg of soil (pH: 7.45; electrical conductivity (EC): 589.66 µS/cm; organic matter (OM): 6.43%; available phosphorus (AP): 2.32%; total nitrogen (TN): 0.084 mg/g; available potassium (AK): 109 mg/kg; calcium carbonate: 11.86%; sand: 79.38%; silt: 7.76%; clay: 3.26%) previously sterilized for 4 h at 300 °C. The seed trays were placed in a controlled greenhouse under the following environmental conditions: temperature of 25/18 °C with a day/night cycle of 16/8h, natural light intensity ranging from 500 to 750 µmol m^−2^ s^−1^, and relative humidity of 60%.

The biostimulant solutions used in this study included three formulations: compost, arbuscular mycorrhizal fungi (AMF), and a combination of compost and arbuscular mycorrhizal fungi. The compost was prepared from green waste [70] and was applied at a rate of 5% relative to soil quantity. The physico-chemical characteristics of compost based on dry matter were: pH: 7.56; EC: 7.78 mS/cm; organic matter (OM): 41.71%; mineral matter (MM) 58.28%; total organic carbon (TOC): 30%; AP: 21.68%; TN: 1.4 mg/g. AMF were isolated from the rhizospheric soil (saline soil) of the date palm of the Tafilalet palm grove of the southern region of Morocco. To ensure the multiplication and the growth of AMF, *Zea mays* was used as a host plant. Tomato plants were inoculated with 20 g of multiplication soil. The same autoclaved amount was added to uninoculated plants. 

In order to examine the effects of the application of the mentioned biostimulants and their combinations on plants under salt stress, a total of 32 seedlings were divided into 8 groups, with each group containing 4 replicates and each pot containing 1 seedling. The following treatments were applied: Ct treatment (untreated plants), C treatment (plants treated with compost), M treatment (plants treated with arbuscular mycorrhizal fungi), and CM treatment (plants treated with the combination of compost and AMF), under 0 and 150 mM of NaCl. Then, the plants were randomly placed in the greenhouse. The transplants were regularly irrigated with distilled water for 20 days after AMF inoculation, then were subjected to two NaCl levels (0 and 150 mM). The concentration of saline solution was gradually increased every week (50 mM the first week, 100 mM the second week, and 150 mM the third week) to avoid osmotic shock. Three months after the application of salt stress, the tomato plants were harvested, and their physiological, morphological, and biochemical parameters were evaluated. 

### 4.2. Growth, Yield, and Mycorrhization Assessment

The following growth and yield performances of tomato plants were evaluated: plant height, number flowers and fruits, as well as shoot and root fresh and dry matter, obtained after drying the samples at 60 °C for 48 h.

To assess mycorrhization, root samples were thoroughly washed with distilled water and treated with 10% KOH at 90 °C for 30 min to remove impurities. After further rinsing, the roots were acidified with 2% hydrochloric acid for 10 min, then stained with Trypan blue at 90 °C for 20 min, as described by Phillips and Hayman [71]. Evaluation of root colonization by AMF was performed using the method of McGonigle et al. [72] for precise microscopic analysis conducted using a Zeiss Axioskop 40 microscope (Zeiss, Burladingen, Germany) at 40–100× magnification. 

### 4.3. Measurement of Photosystem II Photochemical Efficiency and Stomatal Conductance 

Using a portable fluorometer (Opti-Science, OSI 30p), the photochemical efficiency of photosystem II was evaluated. Emissions of minimum fluorescence (F_0_), maximum fluorescence (F_m_), and variable fluorescence (F_v_) were measured on the second-youngest fully developed leaves exposed to light after 20 min of adaptation to darkness using tweezers [73]. F_v_/F_m_ was used to estimate photosystem II photochemical efficiency.

During the period of maximum opening of the stomata between 10:00 a.m. and 12:00 p.m., stomatal conductance (gs) was measured using a porometer (Leaf Porometer, Decagon Device, Inc., Washington, DC, USA). Measurements were made on the abaxial side of the third-youngest fully developed leaf for the plants of each treatment. This parameter is expressed in mmol H_2_O m^−2^ s^−1^ [74]. 

### 4.4. Nitrogen Content

Due the significance of plant N nutrition after AMF and/or compost application, N content was determined using the Kjeldahl method [75]. To achieve this, 0.5 g of the sample was mixed with 5 mL of sulfuric acid and a Kjeldahl catalyst, then subjected to mineralization. This step involved heating the mixture to convert organic nitrogen into mineral nitrogen in the form of ammonium. After mineralization, the mixture was neutralized, and the nitrogen was distilled using a Kjeldahl apparatus (KjelFlex K-360, Shandong, China). The released ammonia was then titrated with 0.1 N sulfuric acid to determine the total nitrogen content in the sample.

### 4.5. Photosynthetic Pigments and Soluble Sugars Content

As described by Arnon [76], the photosynthetic pigments were extracted from samples of frozen leaf powder in 80% acetone, and then the extract was centrifuged at 1000× *g* for 10 min. Supernatant optical density (OD) was recorded at 480, 645, and 663 nm using a spectrophotometer. 

Regarding the determination of total soluble sugar (TSS) content, 100 mg of fresh material was homogenized in 2 mL of 80% ethanol, then centrifuged at 5000 rpm for 10 min. A total of 125 µL of supernatant was recovered and mixed with 125 µL of a 5% phenol solution and 625 µL of concentrated sulfuric acid. After 15 min, the TSS content was measured using a 485 nm OD and calculated using the standard glucose curve [77].

### 4.6. Dosage of Malondialdehyde and Hydrogen Peroxide Content

Malondialdehyde (MDA) content was evaluated using Dhindsa et al.’s [78] method by homogenizing frozen leaf and root powder (100 mg) samples in 1 mL of 10% trichloroacetic acid (TCA) and 1 mL of cold acetone, then centrifuging at 8000× *g* for 15 min. A volume of 250 µL of the supernatant was recovered and added to 0.5 mL of 0.1% phosphoric acid and 0.5 mL of 0.6% thiobarbituric acid (TBA); then, the mixture was incubated at 100 °C for 30 min. The reaction was stopped by placing the tubes in an ice bath, and then 0.75 mL of 1-butanol was added to the mixture. After centrifugation at 8000× *g* for 15 min, the butanolic layer was read at an OD of 532 and 600 nm. The amount of MDA was expressed as µmol/g of fresh matter. 

The hydrogen peroxide (H_2_O_2_) content of leaves and roots was determined using the method of Velikova et al. [79]. A total of 100 mg of plant material was ground in a mortar with 2 mL of 10% TCA and then centrifuged at 12,000× *g* for 10 min. Then, 0.5 mL of supernatant was recovered and added to 0.5 mL of potassium phosphate buffer (10 mM, pH 7) and 1 mL of iodine potassium (1 M). The mixture was vortexed and incubated for three minutes at room temperature and one hour in the dark. The H_2_O_2_ content was measured using a 390 nm OD spectrophotometer. 

### 4.7. Determination of Protein Content and Antioxidant Enzyme Activity

The assessment of leaf and root enzyme activity was carried out according to the method of Tejera García et al. [80]. First, 100 mg of plant material was crushed in the presence of 4 mL of phosphate buffer (0.1 mM, pH 7) containing 5% polyvinylpyrrolidone (PVP) and 0.1 mM ethylenediaminetetraacetic acid (ETDA). The mixture was centrifuged at 18,000× *g* for 10 min, the supernatant was recovered and stored at −20 °C for biochemical assays. Polyphenol oxidase (PPO) activity was determined by the method of Hori et al. [81]. The test solution contained 10 mM catechol in a phosphate buffer (0.1 M, pH 7). The reaction began after adding the enzyme extract. Peroxidase (POX) activity was measured using the method of Polle et al. [82]. The peroxidase POX assay was performed using the guaiacol test (ability to convert guaiacol to tetraiacol following the change in OD at 470 nm). The reaction mixture contained phosphate buffer (0.1 M, pH 7), 20 mM guaiacol, 10 mM H_2_O_2_, and 0.1 mL of enzyme extract. Ascorbate peroxidase (APX) activity was measured using the Nakano and Asada method [83]. The test mixture contained phosphate buffer (100 mM, pH 6), 10 mM ascorbic acid, 200 mM H_2_O_2_, and 0.5 mL of enzyme extract. Ascorbate peroxidase (APX) activity was determined by a decrease in the absorbance at 290 nm for 1 min. Catalase activity was measured by monitoring the decreasing OD at 240 nm for 3 min after decomposition of H_2_O_2_ [84]. The reaction mixture contained a potassium phosphate buffer (100 mM, pH 6), 0.5 mL of enzymatic extract, and 10 mM H_2_O_2_. Total soluble protein content was determined according to the Bradford protocol [85], with bovine serum albumin (BSA) as the standard. 

### 4.8. Statistical Analyses 

The experimental data were presented as mean values with standard error (SE) based on three replicates per treatment. Statistical analyses were conducted using the Costat software package. A three-way randomized block analysis of variance (ANOVA) was performed to assess the main effects (salinity, S; compost, C; AMF inoculation, AMF) and their interactions. Principal component analysis (PCA) was conducted using XLSTAT-software v. 2019 to explore interactions among variables and treatments. Mean comparisons were assessed using Duncan’s test at a significance level of *p* < 0.05.

## 5. Conclusions

Plants are forced to adapt to harsh climatic conditions, like increased soil salinity, which severely reduces their ability to absorb water and nutrients. This leads to a variety of adverse effects on plants. Salinity stress affected the development and growth of tomato crops as well as physiological and biochemical parameters. Applying biostimulants to plants allows them to develop more efficiently and resist stress better. The application of AMF or compost alone or in combination showed positive effects on growth parameters as well as photosynthetic parameters. This application also mitigated oxidative stress by reducing the levels of hydrogen peroxide and malondialdehyde while increasing the activity of antioxidant enzymes such as catalase, polyphenol oxidase, peroxidase, and ascorbate peroxidase. The most efficient treatment to promote tomato tolerance to salt stress was the combination of AMF and compost, followed by AMF applied alone and then compost applied alone. Further investigation related to genomic, transcriptomic, proteomic, and metabolomic aspects are needed to provide more insight into the molecular mechanisms orchestrating tomato’s tolerance to salt stress in the presence of AMF and/or compost.

## Figures and Tables

**Table 1:** Growth and yield traits and AMF infection of tomato plants under saline and non-saline conditions after the application of compost and AMF alone or in combination.

NaCl Levels	0 mM				150 mM			
Treatments	Ct	C	M	CM	Ct	C	M	CM
PH ^(cm)^	74.00 ± 4.58 ^ab^	69.67 ± 4.16 ^bc^	66.83 ± 3.75 ^bcd^	81.00 ± 2.65 ^a^	61.13 ± 6.19 ^cd^	65.30 ± 4.20 ^bcd^	58.00 ± 3.46 ^d^	63.00 ± 6.93 ^cd^
RE ^(cm)^	25.00 ± 1.32 ^b^	33.17 ± 6.33 ^ab^	36.50 ± 7.40 ^a^	28.17 ± 5.84 ^ab^	30.33 ± 0.58 ^ab^	33.70 ± 8.97 ^ab^	31.90 ± 4.15 ^ab^	37.80 ± 6.67 ^a^
NL	72.67 ± 6.43 ^ab^	61.67 ± 7.51 ^bc^	64.33 ± 8.74 ^bc^	51.33 ± 10.02 ^c^	55.67 ± 12.50 ^c^	53.00 ± 7.94 ^c^	84.67 ± 6.66 ^a^	57.33 ± 7.50 ^bc^
NFl	4.33 ± 3.51 ^b^	5.00 ± 2.00 ^b^	5.33 ± 3.21 ^b^	4.67 ± 0.58 ^b^	2.33 ± 0.58 ^b^	3.67 ± 1.15 ^b^	12.33 ± 0.58 ^a^	2.67 ± 3.06 ^b^
NFr	12.67 ± 2.08 ^a^	8.33 ± 2.08 ^bc^	9.00 ± 4.36 ^abc^	7.00 ± 0.00 ^c^	5.33 ± 0.58 ^c^	6.67 ± 0.58 ^c^	8.67 ± 1.15 ^abc^	12.00 ± 3.00 ^ab^
SFW ^(g/plant)^	375.00 ± 12.00 ^ab^	261.00 ± 28.00 ^cd^	347.00 ± 31.00 ^b^	433.00 ± 25.00 ^a^	255.00 ± 26.00 ^cd^	218.00 ± 51.00 ^d^	362.00 ± 44.00 ^b^	310.00 ± 56.00 ^bc^
RFW ^(g/plant)^	8.61 ± 2.24 ^c^	19.10 ± 4.46 ^c^	12.78 ± 1.39 ^c^	12.57 ± 2.32 ^c^	67.83 ± 23.07 ^ab^	65.48 ± 13.30 ^ab^	80.33 ± 16.03 ^a^	57.09 ± 13.01 ^b^
SDW ^(g/plant)^	11.92 ± 1.54 ^ab^	12.40 ± 0.71 ^ab^	11.82 ± 0.83 ^ab^	10.55 ± 0.31 ^b^	12.52 ± 2.11 ^ab^	11.30 ± 0.57 ^ab^	11.14 ± 0.49 ^ab^	12.71 ± 0.35 ^a^
RDW ^(g/plant)^	33.64 ± 1.01 ^a^	28.74 ± 3.29 ^a^	18.08 ± 10.57 ^b^	12.97 ± 2.95 ^b^	11.76 ± 3.32 ^b^	12.99 ± 0.65 ^b^	14.42 ± 4.02 ^b^	16.70 ± 1.94 ^b^
Fv/Fm	0.78 ± 0.01 ^a^	0.75 ± 0.04 ^a^	0.67 ± 0.03 ^bc^	0.36 ± 0.02 ^c^	0.70 ± 0.03 ^b^	0.79 ± 0.02 ^a^	0.75 ± 0.00 ^a^	0.78 ± 0.01 ^a^
gs ^(mmol m−2 s −1)^	172.97 ± 27.00 ^bc^	153.30 ± 26.00 ^c^	286.47 ± 58.00^a^	177.87 ± 70.00 ^bc^	293.00 ± 67.00 ^bc^	173.53 ± 18.00 ^bc^	253.27 ± 50.00 ^ab^	220.13 ± 7.00 ^abc^
Fa (%)	0.00 ± 0.00 ^d^	13.33 ± 23.00 ^d^	68.86 ± 10.00 ^ab^	57.77 ± 25.00 ^bc^	0.00 ± 0.00 ^d^	0.00 ± 0.00 ^d^	82.22 ± 10.18 ^a^	40.00 ± 6.67 ^c^
Ma (%)	0.00 ± 0.00 ^d^	0.02 ± 0.04 ^d^	11.55 ± 3.79 ^b^	4.55 ± 3.17 ^d^	0.00 ± 0.00 ^d^	0.00 ± 0.00 ^d^	15.89 ± 2.41 ^a^	1.33 ± 0.67 ^cd^

C: compost; Ct; control; CM: combination of compost and arbuscular mycorrhizal fungi; M, arbuscular mycorrhizal fungi; PH, plant height; RE: root elongation; NL: number of leaves; NFl: number of flowers; NFr: number of fruits; SFW: shoot fresh weight; RFW: root fresh weight; SDW: shoot dry weight; RDW: root dry weight; gs: stomatal conductance; Fa: AMF infection frequency; Ma: AMF infection intensity. Data are means of three replicates (n = 3) ± standard error (SE). The values of each parameter labeled by different letters indicate significant differences assessed by Duncan’s test after performing a three-way ANOVA (*p* < 0.05).

**Table 2:** Shoot antioxidant enzyme activity of tomato plants under saline and non-saline conditions after the application of compost and AMF alone or in combination.

NaCl Levels	Treatments	CAT (µmol H_2_O_2_/mg prot/min)	PPO (µmol Catéchol/mg prot/min)	APX (µmol Asc/mg prot/min)	POX (µmol Guaiacol/mg prot/min)
0 mM	Ct	130.28 ± 61.90 ^c^	0.38 ± 0.03 ^b^	0.58 ± 0.80 ^a^	0.13 ± 0.01 ^c^
	C	178.48 ± 66.55 ^c^	0.70 ± 0.24 ^b^	1.35 ± 1.07 ^a^	0.36 ± 0.13 ^ab^
	M	178.93 ± 34.57 ^c^	0.59 ± 0.18 ^b^	1.01 ± 0.69 ^a^	0.36 ± 0.10 ^ab^
	CM	208.13 ± 41.43 ^c^	0.67 ± 0.27 ^b^	0.95 ± 0.40 ^a^	0.27 ± 0.17 ^abc^
150 mM	Ct	316.47 ± 73.09 ^b^	0.83 ± 0.12 ^b^	1.86 ± 1.15 ^a^	0.16 ± 0.06 ^bc^
	C	485.23 ± 42.14 ^a^	2.72 ± 0.24 ^a^	2.13 ± 2.84 ^a^	0.37 ± 0.11 ^ab^
	M	516.77 ± 65.72 ^a^	3.26 ± 1.06 ^a^	2.51 ± 2.84 ^a^	0.41 ± 0.19 ^a^
	CM	446.57 ± 85.77 ^a^	2.76 ± 0.74 ^a^	1.12 ± 1.38 ^a^	0.35 ± 0.08 ^abc^

C: compost; Ct: control; CM: combination of compost and arbuscular mycorrhizal fungi; M: arbuscular mycorrhizal fungi; APX: ascorbate peroxidase; CAT: catalase; POX: peroxidase; PPO: polyphenol oxidase. Data are means of three replicates (n = 3) ± standard error (SE). The values of each parameter labeled by different letters indicate significant differences assessed by Duncan’s test after performing three-way ANOVA (*p* < 0.05).

**Table 3:** Root antioxidant enzyme activity of tomato plants under saline and non-saline conditions after the application of compost and AMF alone or in combination.

NaCl Levels	Treatments	CAT (µmol H_2_O_2_/mg prot/min)	PPO (µmol Catechol/mg prot/min)	APX (µmol Asc/mg prot/min)	POX (µmol Guaiacol/mg prot/min)
0 Mm	Ct	264.45 ± 56.00 ^c^	0.52 ± 0.08 ^b^	3.48 ± 1.82 ^b^	3.77 ± 1.66 ^bc^
	C	326.69 ± 69.73 ^c^	0.59 ± 0.05 ^b^	3.58 ± 2.43 ^b^	0.59 ± 0.28 ^d^
	M	287.55 ± 38.39 ^c^	0.63 ± 0.04 ^b^	0.96 ± 0.50 ^b^	1.43 ± 0.80 ^cd^
	CM	439.78 ± 85.32 ^bc^	0.52 ± 0.08 ^b^	4.64 ± 4.59 ^b^	0.97 ± 0.53 ^d^
150 mM	Ct	508.46 ± 158.77 ^b^	1.57 ± 0.72 ^b^	8.26 ± 9.08 ^b^	5.81 ± 2.37 ^ab^
	C	788.46 ± 158.40 ^a^	3.37 ± 1.46 ^a^	19.12 ± 1.60 ^a^	6.77 ± 1.94 ^a^
	M	520.86 ± 40.50 ^b^	0.52 ± 0.13 ^b^	2.57 ± 1.87 ^b^	1.63 ± 0.85 ^cd^
	CM	605.65 ± 104.59 ^b^	0.59 ± 0.19 ^b^	0.89 ± 0.59 ^b^	1.04 ± 0.21 ^d^

C: compost; Ct: control; CM: combination of compost and arbuscular mycorrhizal fungi; M: arbuscular mycorrhizal fungi; APX: ascorbate peroxidase; CAT: catalase; POX: peroxidase; PPO: polyphenol oxidase. Data are means of three replicates (n = 3) ± standard error (SE). The values of each parameter labeled by different letters indicate significant differences assessed by Duncan’s test after performing three-way ANOVA (*p* < 0.05).

## Data Availability

Data are contained within the article and supplementary materials.

## Supplementary Materials

The following supporting information can be downloaded at: https://www.mdpi.com/article/10.3390/plants13162225/s1, Figure S1. Phenotypic effects of tomato plants under saline and non-saline conditions after the application of compost and AMF alone or in combination. Ct; control; C: compost; M, arbuscular mycorrhizal fungi; CM: combination of compost and arbuscular mycorrhizal fungi.
