# Supplementary material for: The Use of Compost and Arbuscular Mycorrhizal Fungi and Their Combination to Improve Tomato Tolerance to Salt Stress
Source: Plants (Basel). 2024 Aug 11;13(16):2225. doi: 10.3390/plants13162225 (PMC11359464; doi:10.3390/plants13162225)
Supplement: Supplementary file 1 [file plants-13-02225-s001.zip › plants-3129895-supplementary.pdf]

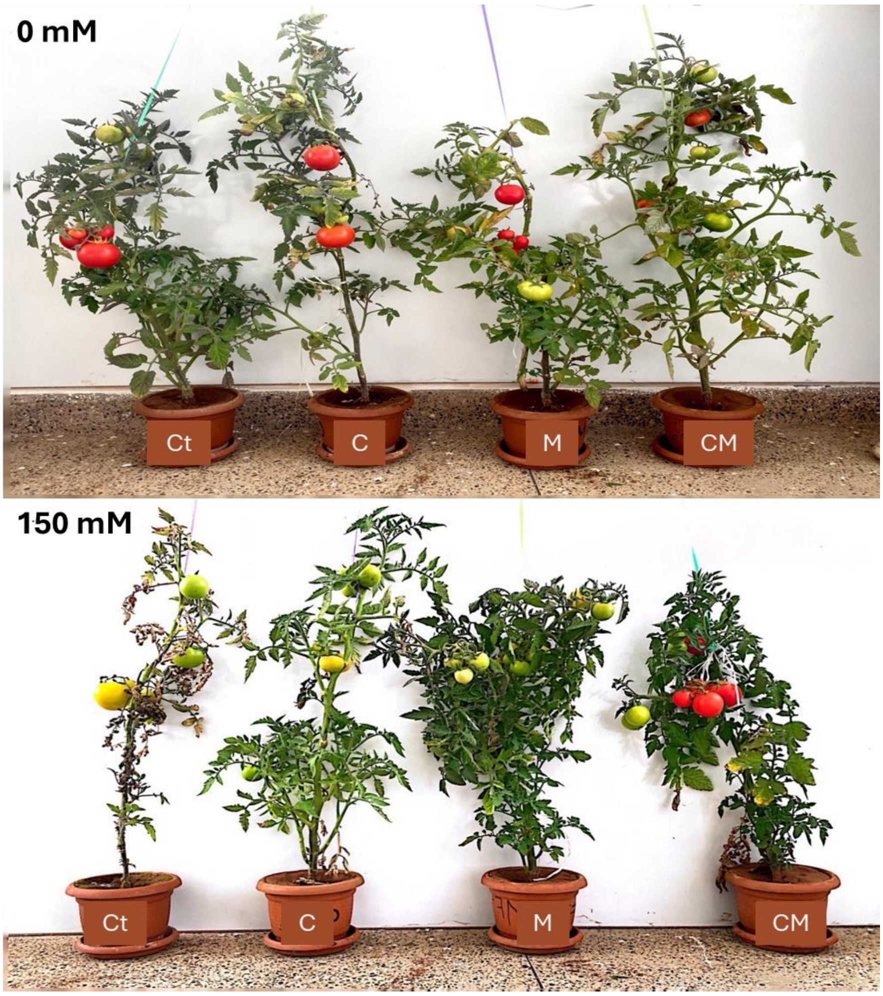

Figure S1. Phenotypic effects of tomato plants under saline and non-saline conditions after the application of compost and AMF alone or in combination. Ct; control; C: compost; M, arbuscular mycorrhizal fungi; CM: combination of compost and arbuscular mycorrhizal fungi.
